# Impact of the COVID-19 pandemic on *Haemophilus influenzae* infections in pediatric patients hospitalized with community acquired pneumonia

**DOI:** 10.1038/s41598-024-62728-2

**Published:** 2024-06-03

**Authors:** Ling Ai, Liang Fang, Beizhong Liu, Chanjuan Zhou, Fang Gong

**Affiliations:** 1 Department of General Practice, Yongchuan Hospital of Chongqing Medical University, No. 439, Xuanhua Street, Chongqing, 402160 China; 2 Department of Respiratory and Critical Care Medicine, The Affiliated Hospital of Southwest Medical University, Luzhou, 646000 Sichuan China; 3 Central Laboratory, Yongchuan Hospital of Chongqing Medical University, Chongqing, 402160 China; 4 Department of Neurology, Yongchuan Hospital of Chongqing Medical University, Chongqing, 402160 China; 5 Key Laboratory of Laboratory Medical Diagnostics, Ministry of Education, Department of Laboratory Medicine, Chongqing Medical University, Chongqing, 400016 China; 6 Department of Pediatrics, Yongchuan Hospital of Chongqing Medical University, Chongqing, 402160 China

**Keywords:** COVID-19, *Haemophilus influenzae*, Community acquired pneumonia, Epidemiology, Antimicrobial resistance, Pediatrics, Microbiology, Infectious diseases

## Abstract

The COVID-19 pandemic has altered the infection landscape for many pathogens. This retrospective study aimed to compare *Haemophilus influenzae* (*H. influenzae*) infections in pediatric CAP patients hospitalized before (2018–2019) and during (2020–2022) the COVID-19 pandemic. We analyzed the clinical epidemiology and antimicrobial resistance (AMR) patterns of *H. influenzae* from a tertiary hospital in southwest China. A total of 986 pediatric CAP patients with *H. influenzae*-associated infections were included. Compared to 2018, the positivity rate increased in 2019 but dropped significantly in 2020. Although it rose in the following 2 years, the rate in 2022 remained significantly lower than in 2019. Patients’ age during the pandemic was significantly higher than in 2018 and 2019, while gender composition remained similar across both periods. Notably, there were significant changes in co-infections with several respiratory pathogens during the pandemic. Resistance rates of *H. influenzae* isolates to antibiotics varied, with the highest resistance observed for ampicillin (85.9%) and the lowest for cefotaxime (0.0%). Resistance profiles to various antibiotics underwent dramatic changes during the COVID-19 pandemic. Resistance to amoxicillin-clavulanate, cefaclor, cefuroxime, trimethoprim-sulfamethoxazole, and the proportion of multi-drug resistant (MDR) isolates significantly decreased. Additionally, MDR isolates, alongside isolates resistant to specific drugs, were notably prevalent in ampicillin-resistant and β-lactamase-positive isolates. The number of pediatric CAP patients, *H. influenzae* infections, and isolates resistant to certain antibiotics exhibited seasonal patterns, peaking in the winter of 2018 and 2019. During the COVID-19 pandemic, sharp decreases were observed in February 2020, and there was no resurgence in December 2022. These findings indicate that the COVID-19 pandemic has significantly altered the infection spectrum of *H. influenzae* in pediatric CAP patients, as evidenced by shifts in positivity rate, demographic characteristics, respiratory co-infections, AMR patterns, and seasonal trends.

## Introduction

Globally, community acquired pneumonia (CAP) remains a profound public health challenge, being a primary cause for hospitalizations and constituting a significant fraction of antimicrobial prescriptions^1^. Children under 5 years old and adults over 65 years old are particularly susceptible^2^. Hospitalized CAP patients, especially those in intensive care settings, exhibit high mortality rate^3–5^. Pathogens with antimicrobial resistance (AMR) pose a heightened threat, escalating the risk of mortality in children infected with multi-drug resistant (MDR) isolates^6,7^. Yet, pediatric CAP treatments often hinge on empirical methods, as timely etiological identification and drug susceptibility testing aren't always feasible.

*Haemophilus influenzae* (*H. influenzae*) frequently instigates CAP in pediatric populations^8^. An understanding of local resistance trends is paramount for effective empiric therapy. As such, monitoring the bacterial epidemiology and AMR patterns of *H. influenzae* remains a priority. With the advent of *H. influenzae* type b (Hib) vaccination, incidences of *H. influenzae* infections, especially invasive diseases caused by Hib, have seen considerable reductions in numerous countries^9^. Nevertheless, *H. influenzae* continues to place a significant isolate on healthcare systems. For a long duration, ampicillin stood as the preferred drug against *H. influenzae* infections. However, there's a growing prevalence of ampicillin-resistant isolates^10–12^. For instance, in China, the resistance rate for *H. influenzae* isolates to ampicillin surged from 19.9% in 2009–2011 to 32.4% in 2013–2014^13^. Alarmingly, child-isolated *H. influenzae* isolates now exhibit resistance to advanced antibiotics like fluoroquinolones, carbapenems, and third-generation cephalosporins^14^. This escalating resistance complicates the management of pediatric CAP triggered by *H. influenzae*.

COVID-19 was initially reported in December 2019 in Wuhan city, a densely populated area of Hubei province, China. The Chinese government classified COVID-19 as a Class B infectious disease and implemented preventive and containment measures typically reserved for Class A infectious diseases on January 20, 2020^15^. A range of non-pharmaceutical interventions, including cordon sanitaire, social distancing, universal symptom surveys, quarantine strategies, and transport restrictions, were then enforced nationwide^16^. These measures proved effective in mitigating the spread of COVID-19^17^. Nonetheless, the epidemiological landscape of numerous pathogens experienced significant transformations during the COVID-19 pandemic. Notably, the seasonal distributions of *H. influenzae* infection changed in this period^18,19^. Meanwhile, the incidence of invasive bacterial diseases caused by *Streptococcus pneumoniae* (*S. pneumoniae*), *H. influenzae*, and *Neisseria meningitidis* significantly dropped^20,21^. Significant reduction was also shown in *H. influenzae* isolates from the respiratory tract of children in 2020 and 2021^22,23^. However, comprehensive studies exploring the AMR patterns of *H. influenzae* from pediatric CAP patients in the context of the COVID-19 pandemic are sparse^24^. Nearly no study has reported the epidemiology and AMR of *H. influenzae* in 2022 (the third year of COVID-19 pandemic).

In light of this, our study endeavors to gauge the repercussions of the COVID-19 pandemic on the clinical epidemiology and AMR patterns of *H. influenzae* in pediatric CAP patients. Recognizing the public health significance of *H. influenzae* infections in children, this study encompasses data from 2018 to 2022, providing insights into AMR patterns concerning ten antibiotics.

## Materials and methods

### Study population

A retrospective analysis was conducted at Yongchuan Hospital of Chongqing Medical University, a tertiary hospital in western Chongqing, covering the period from January 2018 to December 2022. Pediatric patients diagnosed with CAP were identified using a comprehensive approach involving clinical symptoms, chest imaging, and laboratory tests. Expert radiologists assessed the chest imaging to identify pneumonia-related findings such as infiltrates, consolidation, and other relevant abnormalities. The study encompassed patients aged 1 month to 18 years. Exclusion criteria comprised: (1) Pneumonia onset ≥ 48 h post-admission. (2) Chest radiography revealing interstitial infiltrate, alveolar infiltrate, lobar pneumonia, or pleural effusion > 72 h post-admission. (3) Lung infiltrate or interstitial changes suggestive of pulmonary tuberculosis, pulmonary edema, or atelectasis. (4) Patients with incomplete medical records or absent bacterial culture results. All methods were carried out in accordance with relevant guidelines and regulations. All experimental protocols were approved by the ethics committee of the Yongchuan Hospital of Chongqing Medical University (No. 2023-KeLunShen-76). As a retrospective study, the need for informed consent was waived by the ethics committee of the Yongchuan Hospital of Chongqing Medical University.

### Identification and antimicrobial susceptibility testing of *H. influenzae* isolates

Sputum samples were collected upon admission and submitted for microbiological testing following established clinical protocols. For patients unable to expectorate sputum, samples were obtained either from the nasopharynx or via deep suction under negative pressure. Adequate sputum quality was defined as containing ≥ 25 leukocytes and ≤ 10 epithelial cells under low magnification. The samples underwent cultivation on MacConkey, blood, and chocolate agar plates, followed by an incubation for 18–24 h at 37℃ in a 5% CO2 environment. *H. influenzae* was identified using the Vitek-2 Compact automatic system (BioMérieux, France). The minimum inhibitory concentrations (MICs) of *H. influenzae* isolates were determined using ATB identification cards. The tested antibiotics included ampicillin, amoxicillin and clavulanate, cefaclor, cefuroxime, cefotaxime, rifampicin, ofloxacin, tetracycline, chloramphenicol, and trimethoprim-sulfamethoxazole. All tests aligned with the guidelines set by the Clinical and Laboratory Standards Institute (CLSI), which also provided the criteria for susceptibility classification. The nitrocefin-based method detected *H. influenzae*'s β-lactamase, with a color shift to red indicating a positive result. This study exclusively included unique *H. influenzae* isolates, excluding any repeated isolates from the same patient during the same hospitalization episode. *H. influenzae* ATCC49247 served as the quality-control strain. MDR isolate was defined as the resistance to three or more antibiotic classes.

### Identification of respiratory co-infections

Our study investigated respiratory co-infections involving bacteria, *Mycoplasma pneumoniae* (*M. pneumoniae*), and viruses among children with *H. influenzae*-associated CAP. We assessed bacterial co-infections for three specific isolates: *S. pneumoniae*, *Staphylococcus aureus* (*S. aureus*), and *Moraxella catarrhalis* (*M. catarrhalis*). Co-infections were confirmed when sputum specimens tested positive for both *H. influenzae* and another bacterial species. To detect *M. pneumoniae* co-infections, venous blood samples were collected to isolate serum. The presence of *M. pneumoniae* was determined by detecting Immunoglobulin M (IgM) antibodies in the serum, utilizing either an indirect immunofluorescence assay (IFA) or a passive particle agglutination test (Fujirebio, Japan), following the manufacturer’s instructions. For the passive agglutination test, an antibody titer of ≥ 1:160 was considered indicative of a *M. pneumoniae* infection.

Viral co-infections were detected by collecting venous blood or nasopharyngeal swab samples from patients upon admission. Our viral testing panel comprised five primary respiratory viruses: influenza virus A (IVA), influenza virus B (IVB), parainfluenza virus (PIV), respiratory syncytial virus (RSV), and adenovirus (ADV). Serum IgM antibodies targeting these viruses were quantified using IFA for venous blood samples. Simultaneously, nasopharyngeal swab samples underwent analysis using a multiplex direct immunofluorescence assay kit (Diagnostic Hybrids, Athens, Ohio, USA), following established protocols. Identification of viral co-infections relied on positive findings from either serum or nasopharyngeal swab samples.

### Statistical analysis

The normality of quantitative data was assessed using the Kolmogorov–Smirnov test. Data conforming to a normal distribution were expressed as mean ± standard deviation (SD), and group comparisons were conducted using Student’s t-test. Non-normally distributed data were represented as medians and interquartile ranges, and group comparisons were carried out using the Mann–Whitney U test. According to the actual frequency and theoretical frequency, categorical variables were compared by two-tailed chi-square test, Fisher's exact test, or Yates’ continuity corrected chi-square test. Co-infections were analyzed after excluding patients without corresponding pathogenic results. All statistical evaluations were conducted utilizing the GraphPad Prism 9.0 Software (GraphPad Software, Inc., San Diego, CA, USA). *P* < 0.05 was considered to be statistically significant.

### Ethical approval

All procedures performed in these studies involving human participants were approved by the ethics review committee of the Yongchuan Hospital of Chongqing Medical University, Chongqing, China (No. 2023-KeLunShen-76).

### Consent to participate

As a retrospective study, the need for informed consent was waived.

### Consent for publication

As a retrospective study, the need for informed consent was waived.

## Results

### Impact of the COVID-19 pandemic on the positivity rates, demographic characteristics, and respiratory co-infections of *H. influenzae*-associated CAP patients

This retrospective study involved 6115 pediatric patients hospitalized with CAP from January 2018 to December 2022. Among the 5941 non-duplicate respiratory specimens sent for bacterial culture upon admission, 986 *H. influenzae* isolates were isolated from sputum samples for the AMR data analysis. Despite a significant increase in 2019, the positivity rate of *H. influenzae* notably decreased in 2020 compared to 2018 and 2019. The rate rebounded in the subsequent 2 years, but the level in 2022 was still significantly lower than that in 2019. The *H. influenzae*-associated CAP patients had a median age of 14.5 months (interquartile range: 6–36 months) and included 58.8% males. The distribution of *H. influenzae* infections across various age groups was as follows: 41.6% (410 cases) were children under 1 year, 33.0% (325 cases) were children aged 1 to less than 3 years, 22.8% (225 cases) were children aged 3 to less than 6 years, 2.5% (25 cases) were children aged 6 to less than 12 years, and 0.1% (1 case) were children aged 12 years and older. During the COVID-19 pandemic from 2020 to 2022, the median age was notably higher than that in 2018 and 2019, but no significant changes were observed in the gender composition, as shown in Table 1.

**Table 1:** Comparison of positivity rates, demographic characteristics and co-infection patterns in *H. influenzae*-associated patients before and during the COVID-19 pandemic.

Variables	Total	2018	2019	2020	2021	2022
*H. influenzae*-positives [No. (%)]	986 (16.6)	240 (15.9)	333 (20.5) ^**a**^	83 (9.5) ^**a, b**^	186 (17.9)	144 (16.1) ^**b**^
Age (months)	14.5 (6–36)	11 (6–24)	13 (6–31.5)	27 (7–45) ^**a, b**^	22 (8–42) ^**a, b**^	22.5 (7–44.75) ^**a, b**^
Male patients [No. (%)]	580 (58.8)	141 (58.8)	202 (60.7)	52 (62.7)	101 (54.3)	84 (58.3)
Respiratory co-infections [No. (%)]						
*S. pneumoniae*	120 (12.2)	25 (10.4)	38 (11.4)	8 (9.6)	32 (17.2) ^**a**^	17 (11.8)
*S. aureus*	30 (3.0)	6 (2.5)	17 (5.1)	3 (3.6)	1 (0.5) ^**b**^	3 (2.1)
*M. catarrhalis*	51 (5.2)	7 (2.9)	12 (3.6)	6 (7.2)	20 (10.8) ^**a, b**^	6 (4.2)
*M. pneumoniae*	249 (31.1)	61 (32.8)	93 (33.5)	24 (34.3)	49 (30.8)	22 (20.4) ^**a, b**^
IVA	15 (1.5)	3 (1.3)	11 (3.4)	0 (0.0)	0 (0.0) ^**b**^	1 (0.7)
IVB	4 (0.4)	1 (0.4)	2 (0.6)	0 (0.0)	1 (0.5)	0 (0.0)
PIV	36 (3.8)	13 (5.4)	13 (4.0)	2 (2.5)	4 (2.2)	4 (3.1)
RSV	96 (9.9)	28 (11.7)	36 (11.0)	12 (14.8)	12 (6.6)	8 (6.0)
ADV	7 (0.7)	0 (0.0)	6 (1.8)	1 (1.2)	0 (0.0)	0 (0.0)

Continuous variable was presented as the median (25-75th percentiles); *S. pneumoniae*: *Streptococcus pneumoniae*; *S. aureus*: *Staphylococcus aureus*; *M. catarrhalis*: *Moraxella catarrhalis*; *M. pneumoniae*: *Mycoplasma pneumoniae*; IVA: Influenza virus A; IVB: Influenza virus B; PIV: Parainfluenza virus; RSV: Respiratory syncytial virus; ADV: Adenovirus; a: *P* < 0.05 Versus 2018; b: *P* < 0.05 Versus 2019.

The predominant admission symptoms among patients were cough (98.1%) and fever (45.2%), with wheezing present in 26.6% of cases. In bacterial co-infections, the rate of *H. influenzae*-associated CAP patients co-infected with *S. pneumoniae*, *S. aureus*, and *M. catarrhalis* was 12.2%, 3.0%, and 5.2%, respectively. Comparing to pre-pandemic levels, significant changes were observed in 2021, with more *S. pneumoniae* and *M. catarrhalis* co-infections, but fewer *S. aureus* co-infections (*P* < 0.05). Concerning other pathogens, 31.1% of patients were co-infected with *M. pneumoniae*, and 158 patients had viral co-infections, with RSV being the predominant virus. In comparison to 2018 and 2019, *M. pneumoniae* co-infections notably decreased in 2022. Additionally, the rate of IVA co-infections in 2021 was significantly lower than that in 2019 (*P* < 0.05). However, the co-infections of other viruses did not significantly change during the COVID-19 pandemic, as detailed in Table 1.

### Impact of the COVID-19 pandemic on the antibiotic resistance rates of *H. influenzae* isolates from pediatric CAP patients

As shown in Table 2, *H. influenzae* isolates exhibited relatively high resistance rates to ampicillin, trimethoprim-sulfamethoxazole, cefaclor, and cefuroxime. However, all isolates displayed sensitivity to cefotaxime. MDR isolates accounted for 38.4% of the cases. The most prevalent MDR patterns were resistance to aminopenicillins/ cephalosporins/ sulfonamides/ aminopenicillin with β-lactamase inhibitor (n = 136/379, 35.9%) and aminopenicillins/ cephalosporins/ sulfonamides (n = 135/379, 35.6%). In comparison to 2018, there was a significant decrease in the resistance rate to amoxicillin-clavulanate, cefaclor, cefuroxime, trimethoprim-sulfamethoxazole, and the proportion of MDR isolates in 2019. Notable changes were also observed during the COVID-19 pandemic. Specifically, the resistance rates to amoxicillin-clavulanate, cefaclor, and cefuroxime in 2020 were lower than pre-pandemic levels. Furthermore, the resistance rate to trimethoprim-sulfamethoxazole and the proportion of MDR isolates decreased steadily from 2020 to 2022 (*P* < 0.05). However, the resistance rates to other antibiotics showed no significant changes during this period.

**Table 2:** Comparison of resistance rates of *H. influenzae* isolates before and during the COVID-19 pandemic.

Antibiotics	No. (%) of resistant isolates					
	Total (n = 986)	2018 (n = 240)	2019 (n = 333)	2020 (n = 83)	2021 (n = 186)	2022 (n = 144)
Ampicillin	847 (85.9)	210 (87.5)	287 (86.2)	69 (83.1)	157 (84.4)	124 (86.1)
Amoxicillin-clavulanate	189 (19.2)	61 (25.4)	54 (16.2)^**a**^	8 (9.6)^**a**^	42 (22.6)	24 (16.7)
Cefaclor	372 (37.7)	111 (46.3)	120 (36.0)^**a**^	13 (15.7)^**a, b**^	69 (37.1)	59 (41.0)
Cefuroxime	365 (37.0)	107 (44.6)	118 (35.4)^**a**^	13 (15.7)^**a, b**^	69 (37.1)	58 (40.3)
Cefotaxime	0 (0.0)	0 (0.0)	0 (0.0)	0 (0.0)	0 (0.0)	0 (0.0)
Rifampicin	1 (0.1)	1 (0.4)	0 (0.0)	0 (0.0)	0 (0.0)	0 (0.0)
Ofloxacin	2 (0.2)	1 (0.4)	0 (0.0)	1 (1.2)	0 (0.0)	0 (0.0)
Tetracycline	62 (6.3)	18 (7.5)	24 (7.2)	2 (2.4)	10 (5.4)	8 (5.6)
Chloramphenicol	61 (6.2)	17 (7.1)	17 (5.1)	5 (6.0)	11 (5.9)	11 (7.6)
Trimethoprim-sulfamethoxazole	665 (67.4)	183 (76.3)	219 (65.8)^**a**^	52 (62.7)^**a**^	115 (61.8)^**a**^	96 (66.7)^**a**^
MDR isolates	379 (38.4)	120 (50.0)	117 (35.1)^**a**^	17 (20.5) ^**a, b**^	73 (39.2)^**a**^	52 (36.1)^**a**^

a: *P* < 0.05 Versus 2018; b: *P* < 0.05 Versus 2019.

### Comparison of antibiotic resistance between ampicillin-resistant and sensitive isolates, and β-lactamase positive and negative isolates

Considering the high resistance of *H. influenzae* to ampicillin, a comparative AMR analysis between ampicillin-resistant and ampicillin-sensitive isolates was essential. The resistance rates to amoxicillin-clavulanate, cefaclor, cefuroxime, tetracycline, chloramphenicol, trimethoprim-sulfamethoxazole, and the proportion of MDR isolates were significantly higher in ampicillin-resistant group (*P* < 0.05). The resistance rates to other antibiotics were comparable between two groups (Table 3).

**Table 3:** Comparison of antibiotic resistance between ampicillin-resistant and ampicillin-sensitive *H. influenzae* isolates.

Antibiotics	No. (%) of resistant isolates	
	Ampicillin-resistant isolates (n = 847)	Ampicillin-sensitive isolates (n = 139)
Amoxicillin-clavulanate	189 (22.3)	0 (0.0) ****
Cefaclor	371 (43.8)	1 (0.7) ****
Cefuroxime	365 (43.1)	0 (0.0) ****
Cefotaxime	0 (0.0)	0 (0.0)
Rifampicin	1 (0.1)	0 (0.0)
Ofloxacin	2 (0.2)	0 (0.0)
Tetracycline	60 (7.1)	2 (1.4) **
Chloramphenicol	61 (7.2)	0 (0.0) ****
Trimethoprim-sulfamethoxazole	600 (70.8)	65 (46.8) ****
MDR isolates	379 (44.7)	0 (0.0) ****

* *P* < 0.05; ** *P* < 0.01; *** *P* < 0.001; **** *P* < 0.0001.

Remarkably, 83.5% (823/986) of the *H. influenzae* isolates were positive for β-lactamase. The resistance rates to ampicillin, cefaclor, cefuroxime, tetracycline, chloramphenicol, trimethoprim-sulfamethoxazole, and the proportion of MDR isolates were significantly higher among β-lactamase-positive isolates when compared to β-lactamase-negative isolates (*P* < 0.05) (Table 4).

**Table 4:** Comparison of antibiotic resistance of *H. influenzae* isolates between β-lactamase positive and negative groups.

Antibiotics	No. (%) of resistant isolates	
	β-lactamase positive (n = 823)	β-lactamase negative (n = 163)
Ampicillin	822 (99.9)	25 (15.3) ****
Amoxicillin-clavulanate	164 (19.9)	25 (15.3)
Cefaclor	347 (42.2)	25 (15.3) ****
Cefuroxime	340 (41.3)	25 (15.3) ****
Cefotaxime	0 (0.0)	0 (0.0)
Rifampicin	1 (0.1)	0 (0.0)
Ofloxacin	2 (0.2)	0 (0.0)
Tetracycline	60 (7.3)	2 (1.2) **
Chloramphenicol	61 (7.4)	0 (0.0) ****
Trimethoprim-sulfamethoxazole	578 (70.2)	87 (53.4) ****
MDR isolates	354 (43.0)	25 (15.3) ****

* *P* < 0.05; ** *P* < 0.01; *** *P* < 0.001; **** *P* < 0.0001.

### Impact of the COVID-19 pandemic on the seasonal patterns of *H. influenzae* infections and drug resistant isolates

**Figure 1:**
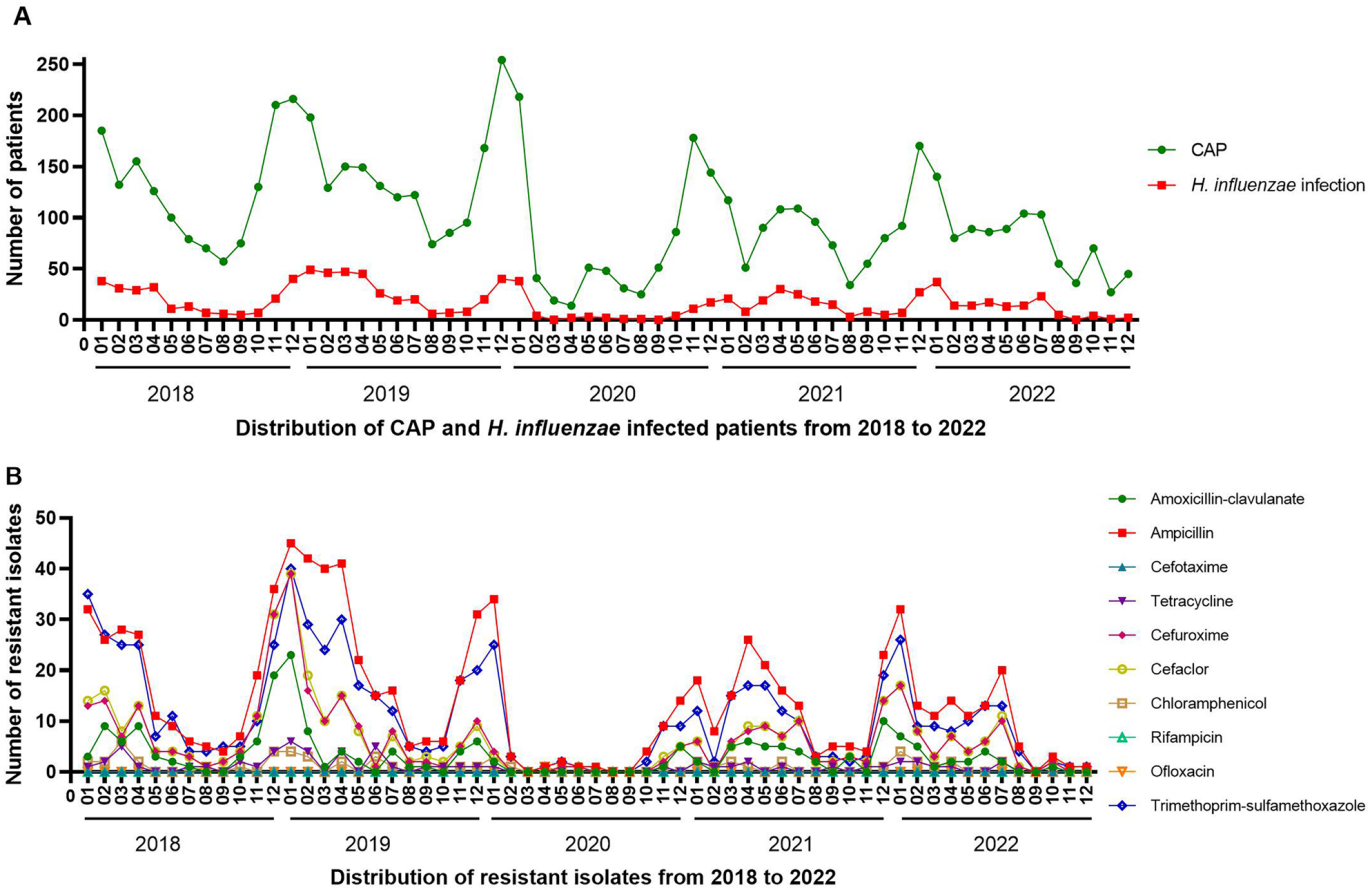
Distributions of pediatric CAP patients, *H. influenzae* isolates, and resistant isolates from January 2018 through December 2022. (**A**) Distribution of CAP and *H. influenzae* infected patients. (**B**) Distribution of resistant isolates.

The number of pediatric CAP patients and *H. influenzae* isolates exhibited seasonal distribution patterns in 2018 and 2019, with increases observed during winter and peaks occurring in December or January. However, the seasonal distributions, particularly those of isolates, underwent shifts during the COVID-19 pandemic. In 2020, there was a significant decrease in February, with isolates absent in March and September, maintaining low levels between April and August. In 2021, *H. influenzae* infections rebounded in April, reaching the highest level for that year. In 2022, isolates were absent in September and then remained at low levels without resurgence (Fig. 1A).

Seasonal trends were also observed in the prevalence of *H. influenzae* isolates resistant to ampicillin, amoxicillin-clavulanate, cefaclor, cefuroxime, and trimethoprim-sulfamethoxazole. Generally, resistance increased during winter months, peaking in December or January, but exhibited lower levels from August to October in both 2018 and 2019. However, these seasonal patterns shifted during the COVID-19 pandemic. Notably, there was a sharp decrease in resistant isolates in February 2020. Moreover, resistant isolates were either absent or maintained at low levels between March and September in 2020, as well as from September to December in 2022. Due to the low resistance rate, the seasonal distribution of *H. influenzae* isolates resistant to other antibiotics remained inconspicuous throughout the five-year period (Fig. 1B).

## Discussion

CAP persists as a dominant contributor to pediatric pneumonia morbidity^25^. The prompt initiation of effective antibiotics remains pivotal in treating pediatric CAP. Continual surveillance of pathogen epidemiology and AMR trends is paramount for informed clinical decision-making, especially with *H. influenzae* being a frequent CAP culprit in children^26^. Our study revealed an 85.9% resistance rate for *H. influenzae* to ampicillin, considerably higher than prior studies^14,26^. The primary mechanism of resistance to ampicillin involves the production of β-lactamase, which hydrolyzes β-lactam antibiotics, rendering them ineffective against bacterial cell wall synthesis^27^. Another mechanism is the mutation of penicillin binding protein 3 (PBP3), which decreases the susceptibility of *H. influenzae* to ampicillin and other β-lactam antibiotics^28^. The β-lactam resistance phenotype mediated by mutation in PBP3 is named β-lactamase-negative ampicillin resistant (BLNAR)^10,26,29^. In our cohort, 83.5% of the *H. influenzae* isolates were β-lactamase positive. But there were 25 BLNAR isolates, revealing a higher positive rate of β-lactamase but lower proportion of BLNAR than previous studies^26,30^. Every BLNAR isolate was resistant to amoxicillin-clavulanate, contrasting with the resistance rate of 20.0% in β-lactamase-positive ampicillin resistant (BLPAR) isolates. This aligns with expectations, given that BLNAR isolates are typically resistant to amoxicillin-clavulanate and ampicillin-sulbactam^26^. While BLPAR isolates are usually sensitive to these β-lactamase-inhibiting compounds^11,13,26^.

Consistent with prior studies^14,26,30,31^, our study noted minimal resistance to rifamycins, fluoroquinolones, and third-generation cephalosporins. *H. influenzae* isolates resistant to fluoroquinolones are generally uncommon in children and have been mainly isolated from older people with chronic lung diseases exposed to frequent quinolone treatments^32–34^. BLNAR isolates with PBP3 mutations or BLPAR isolates may have elevated MIC values of third generation cephalosporins, but the level did not transgress the resistance breakpoint in most isolates^29,35,36^. While rifamycins and fluoroquinolones are infrequently administered to pediatric CAP patients, third-generation cephalosporins emerge as potential treatments for MDR isolates. Nonetheless, vigilant monitoring remains crucial because isolates resistant to third generation cephalosporins have been reported^37,38^.

MDR *H. influenzae* isolates, with their enhanced AMR profiles, pose a growing challenge to pediatric healthcare. In our study, 38.4% of isolates were identified as MDR. The proportion of MDR isolates, and the resistance rates to cefaclor, cefuroxime, tetracycline, chloramphenicol, and trimethoprim-sulfamethoxazole were significantly higher in ampicillin-resistant and β-lactamase-positive isolates. Additionally, we observed prevalence of amoxicillin-clavulanate resistance in ampicillin-resistant isolates and ampicillin resistance in β-lactamase-positive isolates. Ampicillin-resistant isolates from another population has demonstrated same trend of cefuroxime resistance^39^. Our analysis of AMR patterns between β-lactamase-positive and negative isolates parallels the findings of a multicenter study conducted in China^30^. However, disparities in sample sources and study durations have led to divergent results in other populations. For instance, while one study observed a significant prevalence of cefotaxime resistance in β-lactamase-positive isolates^14^, another study reported substantially lower resistance to amoxicillin-clavulanate in this group^40^.

Worldwide, the epidemiological landscape of numerous pathogens underwent discernible shifts due to the COVID-19 pandemic. As compared to pre-pandemic figures, there's a notable decline in invasive infections by *S. pneumoniae*, *H. influenzae*, and *Neisseria meningitidis* in 2020 and 2021^20,21,41–46^. Our study analyzed respiratory infections caused by *H. influenzae* in pediatric CAP patients and revealed dramatic changes during the pandemic. Despite a notable increase in 2019, the positivity rate sharply decreased in 2020, dropping significantly below the levels of 2018 and 2019. Additionally, the rate in 2022 was significantly lower than that in 2019. Similarly, decreased *H. influenzae* infections during the COVID-19 pandemic have also been reported in other pediatric populations^22,23^. In our study, *H. influenzae*-associated CAP patients admitted between 2020 and 2022 were notably older compared to the pre-pandemic period. However, the proportion of males did not significantly change. Similar trends in the ages of children infected with *H. influenzae* during the COVID-19 pandemic have been reported^23^. By contrast, no changes in the distribution of age for most pathogens were observed in another study^47^. Respiratory co-infections can complicate effective treatment and impact prognosis. It has been shown that co-infection of *H. influenzae* and RSV is significantly associated with increased severity of CAP in children and adolescents^48^. In our study, *M. pneumoniae* was the most common pathogen in *H. influenzae* co-infections, affecting 31.1% of CAP patients. Additionally, *S. pneumoniae* and RSV emerged as the most prevalent bacterial and viral co-infections, respectively. This highlights the distinct co-infection patterns of *H. influenzae* among children in the community setting. During the COVID-19 pandemic, decreased viral and bacterial coinfections have been demonstrated in patients with acute respiratory infections^49^. In the *H. influenzae*-associated CAP patients of our study, the co-infections of *S. aureus*, *M. pneumoniae*, and IVA notably decreased. In contrast, we observed significant increases in *S. pneumoniae* and *M. catarrhalis* co-infections, revealing the differential effects of the COVID-19 pandemic on various pathogens.

Notably, the COVID-19 pandemic has also influenced the AMR patterns of *H. influenzae* in our study. A marked decrease in the proportion of MDR isolates and resistance rates to amoxicillin-clavulanate, cefaclor, cefuroxime, and trimethoprim-sulfamethoxazole was observed during this period. Similar trajectories of resistance to cefaclor, cefuroxime, and trimethoprim-sulfamethoxazole have been documented elsewhere^24^. Seasonal fluctuations in *H. influenzae* cases are not uncommon, often peaking during certain months^24,50–52^. Pre-pandemic of COVID-19, our findings pointed to pronounced seasonal distributions for *H. influenzae* infections and resistant isolates, with winter witnessing more cases. However, in line with the findings of two other investigations^18,19^, the seasonal pattern of *H. influenzae* in our study was disrupted following the onset of the COVID-19 pandemic. Few cases of *H. influenzae*-associated CAP and resistant isolates were recorded from April to August in 2020, and from November to December in 2022. Moreover, we observed a complete absence of *H. influenzae* isolates in March and September of 2020, as well as September of 2022. The implementation of non-pharmaceutical interventions against the COVID-19 pandemic likely impeded the respiratory transmission of *H. influenzae* among children.

This study has few limitations. Firstly, the decline in Hib infections due to Hib vaccine proliferation means that non-typeable unencapsulated isolate infections are on the rise^53,54^. These unencapsulated isolates exhibit higher β-lactamase positivity than encapsulated isolates^10^. Unfortunately, our study lacked data to assess changes in Hib vaccination status and *H. influenzae* serotype during the COVID-19 pandemic. Secondly, the absence of detailed clinical data concerning disease severity, treatment modalities, and patient responses limits our ability to conduct a thorough analysis of disease dynamics. Thirdly, drawing samples from a large tertiary hospital might have inadvertently introduced survivorship bias, potentially exaggerating community resistance rates of *H. influenza*. Fourthly, given its single-center and retrospective design, the findings might not be wholly reflective of broader regional patterns.

## Conclusions

In conclusion, this study compared the clinical epidemiology and AMR patterns of *H. influenzae* in pediatric CAP patients during the periods before (2018–2019) and during (2020–2022) the COVID-19 pandemic. The positivity rate for *H. influenzae* notably surged in 2019 but then significantly declined in 2020. Although there was a resurgence in the following two years, the rate in 2022 was significantly lower than that in 2019. In contrast to the significant increases in age during the COVID-19 pandemic, the proportion of male patients remained comparable between the two periods. Significant changes were also observed in the prevalence of co-infections with certain other pathogens, the resistance rates to some antibiotics, and the proportion of MDR isolates. Additionally, the typical seasonal patterns in the number of pediatric CAP patients, *H. influenzae*-infected cases, and resistant isolates shifted to varying degrees. Therefore, a multicenter study involving more participants is essential for ongoing surveillance of *H. influenzae* infections in the post-COVID-19 era.

## Data Availability

The data set used in the study is available from the corresponding author on reasonable request.
